# Targeted next generation sequencing in a young population with suspected inherited malignant cardiac arrhythmias

**DOI:** 10.1038/s41431-017-0060-8

**Published:** 2018-01-17

**Authors:** Anders Krogh Broendberg, Morten Krogh Christiansen, Jens Cosedis Nielsen, Lisbeth Noerum Pedersen, Henrik Kjaerulf Jensen

**Affiliations:** 1 Department of Cardiology, Aarhus University Hospital, Aarhus, Denmark; 2 Department of Clinical Medicine, Health, Aarhus University, Aarhus, Denmark; 3 Department of Molecular Medicine (MOMA), Aarhus University Hospital, Aarhus, Denmark

## Abstract

Aborted sudden cardiac death in the young often is due to inherited heart disease. However, the clinical phenotype in these patients is not always evident. The aim of this study was to identify pathogenic molecular genetic variants in a population with suspected inherited cardiac arrhythmias. Eligible patients were admitted to Aarhus University Hospital, Denmark during the period 1999–2013 with arrhythmias assumed caused by a hereditary heart disease, and in whom no genotype had been established. We used the Danish national pacemaker and ICD registry to identify this cohort. One third (24/80) of the study population had first-line genetic testing with a targeted next-generation sequencing (NGS) panel, and two-third (56/80) of the study population had second-line genetic testing with NGS where prior Sanger sequencing did not reveal a causative variant. Variants were assessed according to the American College of Medical Genetics and Genomics (ACMG) guidelines. We included 80 patients. Median age (IQR) was 38 (28–43) years, 54 (68%) were males. First-line genetic testing identified a genetic variant in 33% (8/24) of the cases and second-line genetic testing revealed a variant in 20% (11/56) of the cases. Eleven variants were considered pathogenic, three likely pathogenic and 10 were variants of unknown significance (VUS). Seventeen variants were very rare with a minor allele frequency (MAF) ≤0.02% in all population databases used in the study. Molecular genetic testing of patients with suspected inherited cardiac arrhythmias with NGS identifies a molecular-genetic cause in a significant proportion of patients.

## Introduction

Sudden cardiac death (SCD) from cardiac arrest accounts for an estimated 15–20% of all deaths [1]. Coronary heart disease and valvular heart disease are the largest contributors to SCD in patients above the age of 35 years, whereas rare inherited cardiac disorders is the primary cause of SCD below the age of 35 years [1].

Rare inherited cardiac disorders are divided into two broad classes; cardiomyopathies and channelopathies. These diseases are mainly considered Mendelian disorders, where a strong monogenic component precipitates the risk of fatal or near fatal arrhythmic events [2].

Genetic channelopathies are caused by mutations in the genes encoding the pore forming subunit of the ion channels (alpha subunit) or the genes encoding the regulatory proteins [3]. Cardiomyopathies are caused by mutations in genes encoding the nucleus, the sarcomeric proteins and the desmosomal proteins causing abnormal myocardium [4].

Until recently the Sanger method has been the gold standard used for DNA sequencing [5]. However, the clinical phenotype in patients with inherited cardiac arrhythmias is not always evident. With the arrival of next-generation DNA sequencing (NGS) it has become possible to sequence all coding regions in a human genome by whole-exome sequencing (WES), the entire genome by whole-genome sequencing (WGS) or targeted sequencing with large gene panels [6, 7].

The aim of the present study was to identify pathogenic variants by first-line molecular genetic testing and re-evaluate patients with second-line molecular genetic testing with a targeted NGS heart panel in a population (≤50 years) with suspected inherited malignant cardiac arrhythmias.

## Methods

### Patient selection

We identified all patients ≤50 years of age treated with an Implantable Cardioverter Defibrillator (ICD) at Aarhus University Hospital from 1999 to 2013 (*n* = 433) using the Danish Pacemaker and ICD registry. Eligibility criteria were assessed by one of the authors (AKB). Patients with congenital heart disease or ischaemic heart disease (IHD) were excluded from the study. All patients with clinically unexplained sustained ventricular tachycardia (VT), ventricular fibrillation (VF) or recurrent malignant syncope were eligible for study enrollment.

### Retrospective clinical evaluation

All study participants have undergone routine blood tests, electrocardiogram, and echocardiogram. Depending on age and clinical presentation, coronary angiography, Holter monitoring, cardiac magnetic resonance imaging (CMR), exercise test, signal-averaged electrocardiogram, electrophysiology study, flecainide challenge test, right heart catheterization, and heart biopsy had been performed to rule out reversible causes of index event.

#### Sanger sequencing analysis

Sanger sequencing analysis was performed in the period 1999–2013. Sanger sequencing analysis was done based on the presumed phenotype, and the following genes was analyzed: arrhythmogenic right ventricular cardiomyopathy (ARVC): *PKP2, DSP, DSG2, DSC2, JUP, TMEM43*, Brugada syndrome (BrS)*: SCN5A*, Catecholaminergic Polymorph Ventricular Tachycardia (CPVT): *RYR2*, dilated cardiomyopathy (DCM): *LMNA*, hypertrophic cardiomyopathy (HCM): *MYBPC3, MYH6, MYH7, TNNI3, TNNT2, TPM1*, Long QT syndrome (LQTS): *KCNQ1, KCNH2, SCN5A, KCNE1, KCNE2*.

### Family evaluation

Recording of family history and family pedigree were conducted in all study participants at the time of study inclusion. Data were uploaded to the Danish national hereditary heart disease web database—Progeny (Progeny Clinical, Progeny Software LLC, USA). Genetic testing of family members (cascade screening) was conducted according to current guidelines [8].

### Next-generation sequencing

Upon study inclusion, all study participants were offered molecular genetic screening with our MOMA heart panel v1 (75 genes). During the study period, the gene panel was upgraded, and all study participants onwards were offered molecular genetic screening with our MOMA heart panel v2 (115 genes). For a full list of genes sequenced, see http://moma.dk/genetic-analysis or Supplemental Table S1 and S2. Potentially pathogenic variants were verified by Sanger sequencing. Genomic DNA was purified from blood, and concentration was measured with Quant-iT Picogreen (Invitrogen, Carlsbad, CA, USA). One microgram was used for TruSeq library preparation according to the manufacturer’s instructions (Illumina, San Diego, CA, USA). Libraries were quantified by KAPA qPCR (KAPA systems, Wilmington, MD, USA). Targeting of the genes was performed using the Nimblegen EZ Choice in solution capture system following the manufacturer’s protocol. Paired-end Sequencing (2 × 150 bp) was performed on the Illumina MiSeq Desktop Sequencer.

### Quantitative analysis

For detection of large genomic deletions or insertions Multiplex Ligation dependent Probe Amplification (MPLA) was performed with the SALSA MLPA probe mix P108-B2 SCN5A P114-B2 long QT syndrome (LQTS), and P168-C1 ARVC (MRC Holland). Results were analyzed with the GeneMapper software (Applied Biosystems), and deviations from two reference samples were assessed by the MAQ-S software (Multiplicom N.V., Niel, Belgium).

### Bioinformatics

Data were imported into the CLC Genomics Workbench 6.0 (Qiagen, Hilden, Germany). Reads were trimmed for low quality bases, ambiguous bases and adaptor sequence followed by mapping to Hg19. After duplicate read removal, variants were called with the probabilistic variant detector requiring a read coverage of at least 30 and a probability of 90. Variants were uploaded to the Cartagenia NGS Bench (Leuven, Belgium) and filtered using the following criteria: all variants were filtered against ExAC, GoNL, 1000 genomes and ESP6500 databases discarding all variants present in >5% in any of these cohorts. Potential splice site variants were kept along with all exonic variants that were non-synonymous.

Alamut Visual (Rouen, France) was used for the assessment of missense, nonsense, splice site variants, and small Indels. All variants were evaluated by: (1) Three different in silico prediction tools (SIFT—http://sift.jcvi.org, Polyphen2—http://genetics.bwh.harvard.edu/pph2, and MutationTaster—http://sift.jcvi.org) to determine probability of variant pathogenicity; (2) association to cardiac disease in three different disease databases (The Humane Gene Mutation Database (HGMD) [9]—http://www.hgmd.org, ClinVar—http://www.ncbi.nlm.nih.gov/clinvar and OMIM—https://www.ncbi.nlm.nih.gov/omim) (3) Minor allele frequency (MAF) in relevant population databases (Exome Sequencing Project (ESP)—http://evs.gs.washington.edu/EVS, the Exome Aggregation Consortium (ExAC)—http://exac.broadinstitute.org/, dbSNP—http://www.ncbi.nlm.nih.gov/dbvar). Finally, all mutations were cross-referenced to a local reference population (*n* = 2000) of whole-exome sequenced Danish individuals. Half of this cohort had type 2 diabetes, while the other half had normal blood sugar levels [10].

### Classification of pathogenicity

Variants were manually assessed and classified as pathogenic (5), likely pathogenic (4), variant of unknown significance (VUS) (3), likely benign (2) or benign (1) according to the Standards and Guidelines for the Interpretation of Sequence Variant: A Joint Consensus Recommendation of the American college of Medical Genetics and Genomics and the Association for Molecular Pathology (ACMG score) [11]. PM2 (ACMG score) were considered fulfilled if MAF in relevant population databases were ≤0.1% according to recommendations [12].

Study data (variants and phenotypes) have been submitted to a freely accessible public database (LOVD database).

### Statistics

Statistical analyses were performed using STATA/IC 13.1 (StataCorp, 4905 Lakeway Dr, College Station, TX 77845, USA). Categorical variables are presented as numbers (percentages) and continuous variables as mean (s.d.) or median (interquartile range (IQR)) as appropriate.

### Ethics

The study was performed in accordance with the Declaration of Helsinki and was approved by the Danish Ethics Committee (record number: 1304077) and the Danish Data Protection Agency. Written consent was obtained from all patients upon inclusion.

## Results

### Patient characteristics

**Fig. 1:**
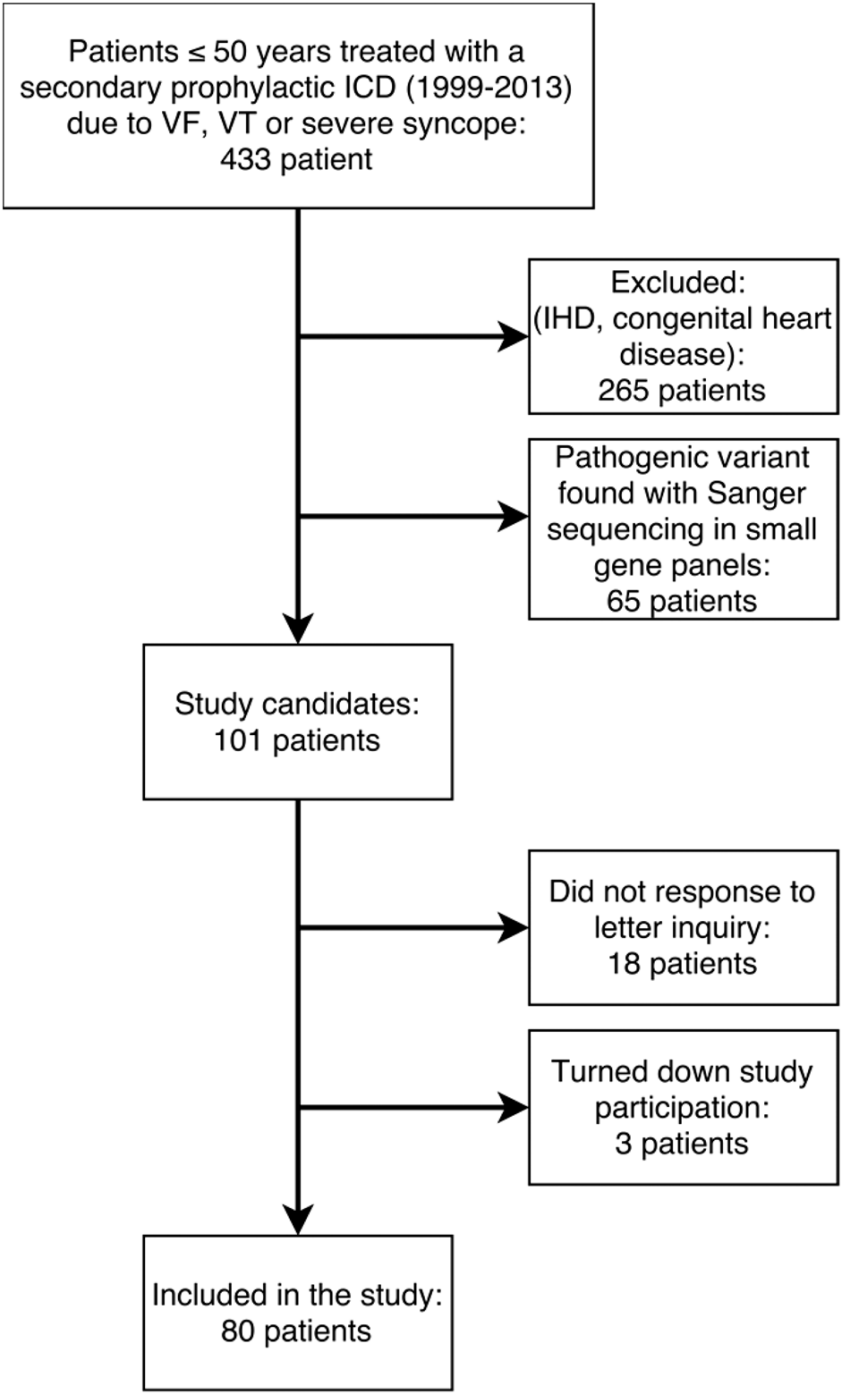
Flow chart showing patient selection in the current study. ICD implantable cardioverter defibrillator; IHD ischemic heart disease, VF ventricular fibrillation; VT ventricular tachycardia

**Fig. 2:**
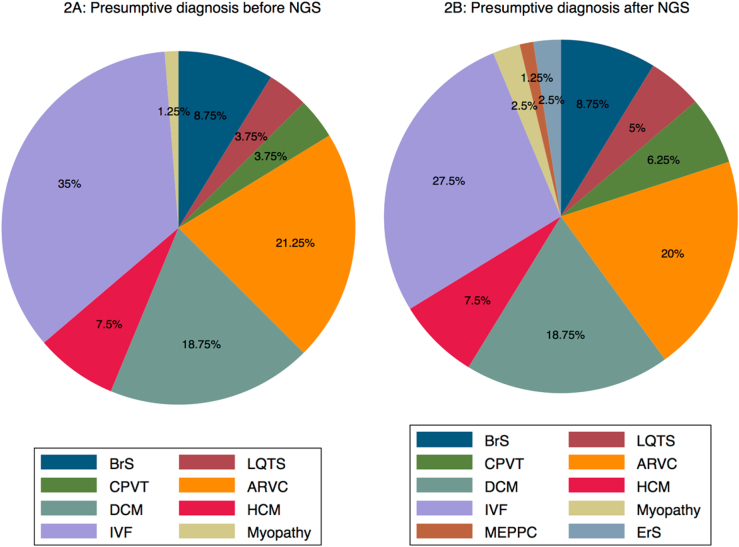
**a**, **b** Presumed phenotype in study cohort before/after re-evaluation with our targeted NGS panel (*n* = 80). ARVC arrhythmogenic right ventricular tachycardia, BrS Brugada Syndrome, CPVT Catecholaminergic polymorph ventricular tachycardia, DCM dilated cardiomyopathy, ErS Early repolarization syndrome, HCM Hypertrophic cardiomyopathy, IVF idiopathic ventricular fibrillation, MEPPC multifocal ectopic premature Purkinje-related contractions

The initial study using Sanger sequencing identified a mutation in 65 patients. Median age (IQR) of this subgroup was 32 (20–39), and 40 (61.5%) were females. Eighty patients (78% of eligible patients) were included in the study and offered targeted NGS screening (Fig. 1). Median age (IQR) of study population was 38 (28–43) (range, 3–49) years, and 54 (68%) were males. At presentation of index event, ventricular fibrillation was observed in 38 (48%) patients; ventricular tachycardia in 33 (41%) patients, and recurrent malignant syncope in 9 (11%) patients (Table 1). In the present study 56 (70%) patients underwent second-line screening with a targeted NGS panel, where prior genetic screening using Sanger sequencing did not reveal a causative genetic variant. The remaining 24 (30%) patients underwent first-line genetic screening with a NGS panel. They had undergone previous clinical evaluation, but were not offered genetic screening in a limited gene panel. Presumed diagnose of the study population before genetic analysis with NGS is shown in Fig. 2a.

**Table 1:** Patient characteristics

Patient characteristics	*N* = 80
Median age (IQR)	38 (28–43) years
Male sex	54 (68%)
*Primary arrhythmia*	
Ventricular fibrillation	38 (48%)
Monomorphic VT	27 (33%)
Polymorphic VT	3 (4%)
Torsades de Pointes VT	3 (4%)
Severe syncope	9 (11%)
*Baseline 12-lead ECG*	
Normal	36 (45%)
Brugada type 1	4 (5%)
Brugada type 2	1 (1%)
Prolonged QTc	4 (5%)
Abnormal T waves^a^	15 (19%)
Abnormal conduction^b^	13 (16%)
Left ventricular hypertrophy	1 (1%)
ST segment deviation	4 (5%)
Premature atrial and ventricular complexes	1 (1%)
Epsilon waves	1 (1%)
*Retrospective clinical evaluation*	
Mean LVEF (range) at admission	53% (10–65%)
Magnetic resonance imaging	41 (51%)
Coronary angiography	60 (75%)
Late potentials	31 (38%)
Flecainide challenge test	7 (9%)
Right heart catheterization	17 (21%)
Holter monitoring	38 (48%)
Exercise test	29 (36%)
Myocardial biopsy	22 (28%)
Electrophysiology study	41 (51%)
- Inducible to VF/VT	18/41 (44%)
*Prospective genetic screening*	
MOMA heart panel v1	10 (13%)
MOMA heart panel v2	70 (87%)
*Family history*	
Probands (n) with SCD in the family	15 (19%)
Probands (n) with aborted SCD in the family	4 (5%)

^a^Inverted T waves, biphasic T waves, ‘camel hump’ T waves, flattened T waves

^b^RBBB, LBBB, 1st, 2nd and 3rd degree AV block, LAH

### Genetic analysis

Molecular genetic testing with Sanger sequencing revealed a possible causative variant in 65 patients (Figure S1–S3). Overall, genetic testing with Sanger sequencing and a targeted NGS panel revealed a possible causative genetic variant (class 3, 4, and 5) in 57% of the total patient cohort (Fig. 1).

Targeted NGS genetic testing revealed 24 possibly disease-causing genetic variants in 19/80 (24%) patients (Tables 2 and 3). All variants were identified in the heterozygote state except a *CRSP3* variant, which was identified in a homozygote state (Table 2). According to the ACMG score 11 variants were considered pathogenic and 3 variants as likely pathogenic, and these variants were considered disease causing (Table 2). Another 10 identified variants were classified as VUS (Table 3). Overall 17 variants had previously been reported to be disease causing, whereas 7 variants were novel. We found 18 missense variants, four deletions and two splice-site variants. Fourteen variants were related to cardiomyopathy and 10 variants were related ion-channel disease. Seventeen variants can be considered very rare with a minor allele frequency (MAF) threshold of ≤0.02% in all used population databases (ESP, ExAC, dbSNP, and 2000 Danes).

**Table 2:** Variants identified by NGS and classified as pathogenic/likely pathogenic according to guidelines

Case	Sex	Age of onset	Phenotype^a^	Gene	Reference sequence	Nucleotide	Amino acid	Coding effect	Minor allele frequency (MAF)				ACMG score	References
									ESP	ExAC	dbSNP	2000 danes		
#1^b^	M	38	DCM/DCM	*RMB20*	(NM_001134363.1)	c.1906C>A	p.(Arg636Ser)	Missense	0%	0%	—	0%	5	Brauch KM [13]
#20^b^	F	12	LQTS/LQTS	*SNTA1*	(NM_003098.2)	c.770C>G	p.(Ala257Gly)	Missense	0.30%	0.33%	0.001%	0.004%	4	Geru W [38]
#23^b^	M	34	ARVC/ARVC	*PKP2*	(NM_004572.3)	c.(336 + 1_337-1)_(1034 + 1_1035-1)del	p.(?)	Deletion	—	—	—	—	5	Novel
#34^b^	F	21	IVF/Myopathy	*NEBL*	(NM_006393.2)	c.180G>A	p.(Lys60Asn)	Missense	—	0.0015%	—	0.0005%	5	Purejav E [39]
#50	M	41	HCM/HCM	*MYBPC3*	(NM_000266.3)	c.772G>A	p.(Glu258Lys)	Splice	—	0.0069%	—	0%	5	Vignier N [40]
#59^b^	M	37	ARVC/ARVC	*PKP2*	(NM_004572.3)	c.(2299 + 1_23001)_(^a^1678…?)	p.(?)	Deletion	—	—	—	—	5	Novel
#62^b^	M	49	IVF/Myopathy	*MYH7*	(NM_000257.2)	c.345 + 1G > A	p.(?)	Splice	—	0.003%	—	—	5	Novel
#63	M	17	IVF/LQTS	*KCNH2*	(NM_000238.3)	c.87 C> A	p.(Phe29Leu)	Missense	—	—	—	0%	4	Kapplinger JD [41]
#75^b^	M	10	ARVC/CPVT	*RYR2*	(NM_001035.2)	c.14553C > A	p.(Phe4851Lys)	Missense	—	—	—	0%	5	Hayashi M [42]
#76^b^	F	33	ARVC/CPVT	*RYR2*	(NM_001035.2)	c.1258C > T	p.(Arg420Trp)	Missense	—	0.0015%	0%	0%	5	Bauce B [43]
#77^b^	F	19	ARVC/MEPPC	*SCN5A*	(NM_198056.2)	c.638G > A	p.(Gly213Asp)	Missense	—	—	—	—	5	Haas J [44]
#78	F	18	IVF/IVF	*CRSP3*	(NM_003476.3)	c.44 del (h)	p.(Lys15fs)	Deletion	—	—	—	—	5	Novel
				*MYL3*	(NM_000258.2)	c.170C > A	p.(Ala57Asp)	Missense	—	0.0075%	0%	0.0005%	4	Kazmierczak K [45]
#79	F	42	IVF/ARVC	*DSP*	(NM_004415.2)	c.(273 + 1_274-1)_(2130 + 1_2131-1)del	p.(?)	Deletion	—	—	—	—	5	Novel

*ARVC* arrhythmogenic right ventricular tachycardia, *CPVT* catecholaminergic polymorph ventricular tachycardia, *DCM* dilated cardiomyopathy, *HCM* hypertrophic cardiomyopathy, *IVF* idiopathic ventricular fibrillation, *LQTS* Long QT syndrome, *MEPPC* multifocal ectopic premature purkinje-related contractions, ?—precise localization of the deletion is unknown and the amino-acid change cannot be predicted

^a^Presumed phenotype before/after investigation with a targeted NGS panel

^b^Previously sequenced in a small gene  panel

*Del* deletion, *(h)* homozygote, (—)—variant not represented in the population database

**Table 3:** Variants identified by NGS and classified as variants of unknown significance according to guidelines

Case	Sex	Age of onset	Phenotype^a^	Gene	Reference sequence	Nucleotide	Amino acid	Coding effect	Frequency population databases (MAF)				ACMG score	References
									ESP	ExAC	dbSNP	2000 danes		
#4	M	49	Myopathy/ARVC	*DSC2*	(NM_004949.3)	c.2194 T > G	p.(Leu732Val)	Missense	0.13%	0.20%	0.001%	0.004%	3	Bhuiyan ZA [27]
				*DSG2*	(NM_001943.3)	c.1174 G > A	p.(Val392Ile)	Missense	0.21%	0.26%	0.001%	0.005%	3	Bhuiyan ZA [27]
				*DSC2*	(NM_004949.3)	c.799 G > A	p.(Ala267Ser)	Missense	—	—	—	0%	3	Novel
#5	M	37	ARVC/ARVC	*DSG2*	(NM_001943.3)	c.137 G > A	p.(Arg46Gln)	Missense	0%	0%	—	0%	3	Awad MM [46]
#15^b^	M	33	IVF/IVF	*SCN5A*	(NM_198056.2)	c.2291 T > G	p.(Met764Arg)	Missense	—	—	—	0%	3	Kapplinger JD [47]
#17	M	48	DCM/DCM	*MYH7*	(NM_000257.2)	c.4660 G > C	p.(Gly1554Gln)	Missense	0.02%	0.003%	—	0%	3	Novel
#72	M	20	IVF/ErS	*ANK2*	(NM_001148.4)	c.11791 G > A	p.(Glu3931Lys)	Missense	0.42%	0.44%	0.001%	0.007%	3	Broendberg AK [48]
#73^b^	M	28	IVF/ErS	*ANK2*	(NM_001148.4)	c.9854 T > C	p.(Ile3285Thr)	Missense	0.9%	1.14%	0.004%	0.02%	3	Broendberg AK [48]
#76^b^	F	33	ARVC/CPVT	*RYR2*	(NM_001035.2)	c.3407 C > T	p.(Ala1136Val)	Missense	1.30%	1.14%	0.002%	0.03%	3	Krahn AD [49]
#78	F	18	IVF/IVF	*KCNE2*	(NM_172201.1)	c.170 T > C	p.(Ile57Thr)	Missense	0.02%	0.11%	0.001%	0.001%	3	Wu J [50]

*ARVC* arrhythmogenic right ventricular tachycardia, *DCM* dilated cardiomyopathy, *ErS* Early repolarization syndrome, *IVF* idiopathic ventricular fibrillation

^a^Presumed phenotype before/after investigation with a targeted NGS panel

^b^Previously sequenced in small gene panels.

(—)—variant not represented in the population database

A possible causative variant (ACMG score 3–5) was found in 11 (20%) study participants, who were offered second-line molecular genetic testing with a NGS panel, and in 8 (33%) study participants offered first-line molecular genetic testing with a NGS panel. Molecular genetic testing with a targeted NGS panel and renewed clinical evaluation changed the diagnosis of 11 patients (Fig. 2a, b and Tables 2 and 3). In addition, genetic re-evaluation confirmed the diagnosis in four patients who had previously been screened negative using a limited gene panel. We found a *RBM20* variant (#1, Table 2), which is a new gene annotated in 2009 and associated with early in life end stage heart failure, need for accelerated heart transplantation and early SCD [13]. *SNTA1* (#20, Table 2) is a rare LQTS gene, which has not previously been part of routine LQTS screening. We also found two large *PKP2* deletions (#23, #59, Table 2) using MLPA technique.

In the current study, 7 (9%) of the participants were associated with a BrS phenotype, and all of them were previously tested in the *SCN5A* gene. Genetic re-evaluation did not reveal any disease-associated variants in the rare BrS genes in these patients. Furthermore, none of the patients with idiopathic ventricular fibrillation were diagnosed with BrS.

### Proband and family cases

In the following section, two cases are selected to illustrate the clinical and genetic difficulties in daily clinical practice, and how cascade screening may add to the judgment of the pathogenicity of identified variants. The clinical presentation of the remaining cases (ACMG score 4 and 5) and the family history are described in the Supplementary Material.

### *RBM20* (NM_001134363.1): c.1906C>A p.(Arg636Ser)

A 38-year old male (#1, Table 2) (pedigree, Figure S4) presented with sustained VT. Echocardiography demonstrated severely dilated cardiomyopathy (DCM) with left ventricular end diastolic diameter of 82 mm and left ventricular ejection fraction (LVEF) of 20%. Molecular genetic testing revealed a *RMB20* variant, which has previously been reported as pathogenic [13]. Family evaluation revealed a severe clinical course of verified DCM (LVEF (range) 15–45%) in six family members in whom the *RBM20* variant was present. Additionally, three family members had a normal echocardiography despite carrying the same variant. The proband and his cousin both underwent heart transplantation. The mother and the son of the proband have clinical DCM with a LVEF of 25% and 35%, respectively, but neither of them has accepted genetic testing. Three family members had died at young ages of 31, 32, and 38 years, respectively, and in all three cases autopsy revealed a dilated left ventricle.

### *DSG2* (NM_001943.3): c.137G>A p.(Arg46Gln)

A 37-year-old man (#5, Table 3; pedigree, Figure S5) was admitted to the hospital with fast sustained VT (210 beats/min). He was treated with synchronized electrical cardioverson to regain sinus rhythm. Echocardiography demonstrated an LVEF of 60%, but paradox movement of the ventricular septum. The right ventricle (RV) was found to be aneurysmatic, dilated (44 mm) and with increased trabeculation. He had an exercise test without arrhythmia and positive late potentials. Cardiac magnetic resonance imaging (CMR) demonstrated a dilated and aneurysmatic RV. He was given a secondary prophylactic ICD.

Molecular genetic testing revealed a rare known *DSG2* variant (Table 3). A brother and a cousin were found to have ARVC according to the 2010 TFC criteria and they were harboring the same *DSG2* variant. They were both given an ICD. Seven additional family members were tested negative for the *DSG2* variant.

## Discussion

SCD in children and young adults is a tragic event, and the search for underlying causes has always attracted attention [14, 15]. A certain percentage of these deaths are due to rare Mendelian diseases. Until now the molecular genetic diagnostic tools, in patients with SCD or survivors after cardiac arrest, have targeted the presumed phenotype, which can be very difficult to define. This means that important genetic information is uncovered inadequately, thus preventing proper counseling and tracing of family members with the same high risk.

In the present study, we found a molecular genetic variant in one fourth of the study population. We identified a significant proportion of possible pathogenic variants by both first-line and second-line molecular genetic screening with a targeted NGS panel.

A new diagnosis was established in 11 participants by genetic testing with a targeted NGS gene panel and subsequent renewed clinical evaluation. Furthermore, renewed genetic re-evaluation confirmed the phenotype in four participants previously genetically tested. The *RBM20* gene and the *SNTA1* gene have not previously been part of routine screening for DCM and LQTS. Second-line testing with a large gene panel might identify rare variants not identified with Sanger sequencing. These results highlight the possible benefit of screening patients with NGS technology. We also found two large deletions in the *PKP2* gene and one large deletion in the *DSP* gene. The patients with a large *PKP2* deletion have previously been assessed with our limited ARVC gene panel. This underlines that adding MPLA technique is essential to correctly identify large molecular genetic re-arrangements associated with disease. Identification of relatives with the same high risk of adverse event is paramount and treatment with anti-arrhythmic medication or an ICD may prevent SCD in the future.

The additional amount of genetic information acquired with the use of NGS is overwhelming. Despite the increase in number of identified pathogenic variants, the interpretation of the molecular genetic findings may be even more difficult, as the number of truly harmless variants (with unknown functions) also increases [6, 16, 17]. This is especially true when using NGS as first-line genetic testing. However, due to an uncertain phenotype in some patient’s preliminary genetic testing with limited gene panel might not be truly possible. The use of a broad gene panel might be beneficial as first-line testing although the trade-off is an increased number of VUS. The major advantage of first-line NGS testing is the possibility of reverse phenotyping, where renewed clinical evaluation can establish a correct geno–phenotype correlation [18]. The issue about signal-to-noise ratio has been scrutinized in different studies. A high prevalence of genetic variants in the human exome sequencing project (ESP) previously associated with LQTS, BrS, and CPVT has been reported [19–21]. Refsgaard et al. [21] sequenced four variants associated with LQTS in a reference population (*n* = 704) and the prevalence was comparable to the ESP data. Further sequencing of four variants associated to BrS in a Danish reference population (*n* = 536) revealed a surprisingly high prevalence (1:30) [20]. Similar results were also found in genes associated with cardiomyopathies [22]. These findings probably indicate that other factors than genetics contribute significantly to disease susceptibility. The present study is based on a strong Mendelian genetic model. However, the monogenic paradigm, where a single gene causes disease has been questioned recently. Some diseases will be classified as near-Mendelian where a strong monogenic component and few genetic modulators cause disease. Other diseases will be more oligogenic (e.g., BrS) [2]. LQTS has a strong Mendelian trait, however, several reports have corroborated that common *NOS1AP* SNPs cause a prolongation of the QTc interval [23, 24]. Furthermore, a GWAS study in 312 BrS patients compared with 1115 controls found that common variants in *SCN10A* and *HEY2* are associated with BrS [25]. The heterogeneity of the BrS phenotype and the rarity of the minor BrS variants are also demonstrated in the current study, as no genotype was established in patients under suspicion of BrS with an extended gene panel.

Interpretation of missenses variants in genes associated with ARVC (*PKP2, DSP, DSG2, DSC2* and *TMEM43*) can be difficult as 16% of healthy controls harbors such a missense variant [26]. However, in case #5 the reported variant (*DSG2*, p.(Arg46Gln)) is situated in a mutational hotspot [26], and is not present in any of the population databases. Furthermore, two other family members with clinically diagnosed ARVC carry this variant, which may imply causality. In case #4 the combination of (*DSC2*, p.(Leu732Val)) and (*DSG2*, p.(Val392Ile)) has proven to be pathogenic [27]. Of note, compound and digenic heterozygosity precipitates to the disease phenotype and these patients have an increased risk of arrhythmic events and SCD [28, 29].

Several other factors contribute to the difficulty in interpreting causality of rare variants. This includes reduced penetrance, variable expressivity and missing information of co-segregation in the family tree.

Furthermore, the conception of channelopathies as having structural normal hearts has also been challenged in the last couple of years. Recently, a new disease entity has emerged named Multifocal Ectopic Premature Purkinje-Related Contractions (MEPPC) associated with atrial and ventricular premature contractions and DCM [30, 31]. Proband #77 was initially suspected for ARVC, however she never fulfilled the 2010 TFC criteria. Molecular genetic testing revealed a rare *SCN5A* variant. *SCN5A* variants have been associated with ARVC in 2% of cases [32]. Renewed clinical evaluation of the proband revealed a high burden of atrial and ventricular premature contractions, and cascade screening revealed multiple affected family members (Supplementary Material, page 13). Furthermore, *RYR2* variants have also been associated with ARVC with an estimated prevalence of 9% [33]. However, renewed clinical evaluation of case 75 and 76 concluded they did not fulfill the 2010 TFC criteria and they had classical CPVT (Supplementary Material, page 12 and Broendberg et al. [34]). Finally, CMR studies in patients diagnosed with BrS have elucidated functional and morphological alterations in the right ventricle compared to matched controls [35, 36].

In 76% of the study population, we found no underlying molecular-genetic cause of their malignant arrhythmic events. This underlines the yet uncovered genetic complexity and environmental interplay of inherited cardiac disorders, which were previously assumed to be monogenic [1]. A broader molecular genetic screening with WES or WGS might elucidate areas in the genome not yet annotated as genes, which could be responsible for some of the study participants’ current phenotype. According to the ENCODE project 80% of the genome might have a regulatory effect [37]. This opens a wealth of possible underlying genetic causes of aborted SCD in the young. However, this is not within the scope of the current project.

Several issues remain regarding interpretation of genetic variants in the era of NGS technology. Clinicians should be solicitous about interpretation of genetic variants due to a possible high signal-to-noise ratio when using large gene panels. Awareness of previous literature, clinical findings, family history, type of mutation, in silico predictions software and MAF in population databases is of utmost importance when validating variants. The ACMG score incorporates all these elements, but nevertheless the clinical reality is not always completely evident. Validation of possible pathogenic variants will remain a challenge in families with reduced penetrance and private mutations.

## Conclusion

A possible pathogenic genetic variant was identified in 33% by first-line genetic screening and in 20% by second-line genetic screening. Our study suggests that genetic screening using a targeted NGS panel, may identify a molecular-genetic cause in a significant proportion of patients with suspected inherited heart disease.

### Limitations

The sample size in the present study is moderate, and the phenotype of included patients is very heterogenic. However, this is a true reflection of the diverse composition of this population in daily clinical work. The study is a retrospectively study with the inherent limitations of this design. Understanding of inherited cardiac diseases and potential arrhythmic triggers has progressed immensely since 1999. Functional studies should be performed for all detected variants to truly establish if the detected variant is disease causing. This has previously been performed for some of the variants detected, but not for all. The ACMG score incorporates a vast number of elements to establish pathogenicity of the variant, but the score has not been validated in large study cohorts.

## Electronic supplementary material

Supplemental File
