# Supplementary material for: Targeted next generation sequencing in a young population with suspected inherited malignant cardiac arrhythmias
Source: Eur J Hum Genet. 2018 Jan 17;26(3):303–13. doi: 10.1038/s41431-017-0060-8 (PMC5838968; doi:10.1038/s41431-017-0060-8)
Supplement: Supplementary file 1 — Supplemental File [file 41431_2017_60_MOESM1_ESM.docx]

**SUPPLEMENTAL**

| ***Table S1 -*** MOMA heart panel v1 (75 genes) | | | |
| --- | --- | --- | --- |
| **Gene** | [**Gene name**](http://moma.dk/genetic-analysis/moma-ngs-gene-panels) | **NM number** | **Location** |
| ABCC9 | ATP-binding cassette, sub-family C (CFTR/MRP), member 9 | NM_005691.2 | 12p12.1 |
| ACTA2 | actin, alpha 2, smooth muscle, aorta | NM_001613.2 | 10q23.31 |
| ACTC1 | actin, alpha, cardiac muscle 1 | NM_005159.4 | 15q14 |
| ACTN2 | actinin, alpha 2 | NM_001103.2 | 1q42q43 |
| AKAP9 | A kinase (PRKA) anchor protein (yotiao) 9 | NM_005751.4 | 7q21-q22 |
| ANK2 | ankyrin 2, neuronal | NM_001148.4 | 4q25-q26 |
| APOB | apolipoprotein B | NM_000384.2 | 2p24-p23 |
| CACNA1C | calcium channel, voltage-dependent, L type, alpha 1C subunit | NM_000719.6 | 12p13.3 |
| CACNB2 | calcium channel, voltage-dependent, beta 2 subunit | NM_201590.2 | 10p12 |
| CALM1 | calmodulin 1 (phosphorylase kinase, delta) | NM_006888.4 | 14q32.11 |
| CALR3 | calreticulin 3 | NM_145046.4 | 19p13.11 |
| CASQ2 | calsequestrin 2 (cardiac muscle) | NM_001232.3 | 1p13.1 |
| CAV3 | caveolin 3 | NM_033337.2 | 3p25 |
| CSRP3 | cysteine and glycine-rich protein 3 (cardiac LIM protein) | NM_003476.3 | 11p15.1 |
| CTF1 | cardiotrophin 1 | NM_001330.3 | 16p11.2 |
| DES | desmin | NM_001927.3 | 2q35 |
| DSC2 | desmocollin 2 | NM_004949.3 | 18q12.1 |
| DSG2 | desmoglein 2 | NM_001943.3 | 18q12.1 |
| DSP | desmoplakin | NM_004415.2 | 6pter-p21 |
| DTNA | dystrobrevin, alpha | NM_032978.6 | 18q12 |
| EYA4 | eyes absent homolog 4 (Drosophila) | NM_004100.4 | 6q23 |
| FBN1 | fibrillin 1 | NM_000138.4 | 15q21.1 |
| FKTN | fukutin | NM_001079802.1 | 9q31-q33 |
| GJA5 | gap junction protein, alpha 5, 40kDa | NM_005266.6 | 1q21.1 |
| GLA | galactosidase, alpha | NM_000169.2 | Xq21.3-q22 |
| GPD1L | glycerol-3-phosphate dehydrogenase 1-like | NM_015141.3 | 3p22.3 |
| JPH2 | junctophilin 2 | NM_020433.4 | 20q12-q13.11 |
| JUP | junction plakoglobin | NM_021991.2 | 17q21 |
| KCNA5 | potassium voltage-gated channel, shaker-related subfamily, member 5 | NM_002234.2 | 12p13 |
| KCNE1 | potassium voltage-gated channel, Isk-related family, member 1 | NM_000219.4 | 21q22.1-q22.2 |
| KCNE2 | potassium voltage-gated channel, Isk-related family, member 2 | NM_172201.1 | 21q22.1 |
| KCNH2 | potassium voltage-gated channel, subfamily H (eag-related), member 2 | NM_000238.2 | 7q36.1 |
| KCNJ2 | potassium inwardly-rectifying channel, subfamily J, member 2 | NM_000891.2 | 17q24.3 |
| KCNQ1 | potassium voltage-gated channel, KQT-like subfamily, member 1 | NM_000218.2 | 11p15.5 |
| KCNQ4 | potassium voltage-gated channel, KQT-like subfamily, member 4 | NM_04700.3 | 1p34 |
| LAMP2 | lysosomal-associated membrane protein 2 | NM_002294.2 | Xq24-q25 |
| LDB3 | LIM domain binding 3 | NM_001080114.1 | 10q22.3-q23.2 |
| LDLR | low density lipoprotein receptor | NM_000527.4 | 19p13.2 |
| LMNA | lamin A/C | NM_170707.2 | 1q22 |
| MYBPC3 | myosin binding protein C, cardiac | NM_000256.3 | 11p11.2 |
| MYH6 | myosin, heavy chain 6, cardiac muscle, alpha | NM_002471.3 | 14q11.2-q13 |
| MYH7 | myosin, heavy chain 7, cardiac muscle, beta | NM_000257.2 | 14q11.2-q13 |
| MYL2 | myosin, light chain 2, regulatory, cardiac, slow | NM_000432.3 | 12q24.11 |
| MYL3 | myosin, light chain 3, alkali; ventricular, skeletal, slow | NM_000258.2 | 3p |
| MYOZ2 | myozenin 2 | NM_016599.3 | 4q26-q27 |
| NEXN | nexilin (F actin binding protein) | NM_144573.3 | 1p31.1 |
| NPPA | natriuretic peptide A | NM_006172.3 | 1p36.21 |
| PCSK9 | proprotein convertase subtilisin/kexin type 9 | NM_174936.3 | 1p34.1-p32 |
| PKP2 | plakophilin 2 | NM_004572.3 | 12p11 |
| PLN | phospholamban | NM_002667.3 | 6q22.1 |
| PRKAG2 | protein kinase, AMP-activated, gamma 2 non-catalytic subunit | NM_016203.3 | 7q35-q36 |
| RBM20 | RNA binding motif protein 20 | NM_001134363.1 | 10q25.3 |
| RYR2 | ryanodine receptor 2 (cardiac) | NM_001035.2 | 1q43 |
| SCN1B | sodium channel, voltage-gated, type I, beta subunit | NM_001037.4 | 19 |
| SCN4B | sodium channel, voltage-gated, type IV, beta subunit | NM_174934.3 | 11q23.3 |
| SCN5A | sodium channel, voltage-gated, type V, alpha subunit | NM_198056.2 | 3p21 |
| SGCD | sarcoglycan, delta (35kDa dystrophin-associated glycoprotein) | NM_000337.5 | 5q33-q34 |
| SLC25A4 | solute carrier family 25 (mitochondrial carrier; adenine nucleotide translocator), member 4 | NM_001151.2 | 4q35 |
| SNTA1 | syntrophin, alpha 1 | NM_003098.2 | 20q11.2 |
| TAZ | tafazzin | NM_000116.3 | Xq28 |
| TCAP | titin-cap | NM_003673.3 | 17q12 |
| TGFB3 | transforming growth factor, beta 3 | NM_003239.2 | 14q24 |
| TGFBR2 | transforming growth factor, beta receptor II (70/80kDa) | NM_003242.5 | 3p22 |
| TMEM43 | transmembrane protein 43 | NM_024334.2 | 3p25.1 |
| TMPO | thymopoietin | NM_003276.2 | 12q22 |
| TNNC1 | troponin C type 1 (slow) | NM_003280.2 | 3p21.1 |
| TNNI3 | troponin I type 3 (cardiac) | NM_000363.4 | 19q13.4 |
| TNNT2 | troponin T type 2 (cardiac) | NM_000363.4 | 1q32 |
| TPM1 | tropomyosin 1 (alpha) | NM_001018005.1 | 15q22.1 |
| TTN | titin | NM_003319.4 | 2q31 |
| TTR | transthyretin | NM_000371.3 | 18q12.1 |
| VCL | vinculin | NM_014000.2 | 10q22.1-q23 |

| ***Table S2 -*** MOMA Heart panel v2 (115 genes)^[[1]](#footnote-1)^ | | | |
| --- | --- | --- | --- |
| **Gene** | [**Gene name**](http://moma.dk/genetic-analysis/moma-ngs-gene-panels) | **NM number** | **Location** |
| ANKRD1 | ankyrin repeat domain 1 (cardiac muscle) | NM_014391.2 | 10q23.31 |
| BAG3 | BCL2-associated athanogene 3 | NM_004281.3 | 10q25.2-q26.2 |
| CACNA2D1 | calcium channel, voltage-dependent, alpha 2/delta subunit 1 | NM_000722.2 | 7q21-q22 |
| CALM2 | calmodulin 2 (phosphorylase kinase, delta) | NM_001743.4 | 2p21 |
| CALM3 | calmodulin 3 (phosphorylase kinase, delta) | NM_005184.3 | 19q13.2-q13.3 |
| CRYAB | crystallin, alpha B | NM_001885.2 | 11q23.1 |
| DMD | dystrophin | NM_004006.2 | Xp21.2 |
| EMD | emerin | NM_000117.2 | Xq28 |
| FBN2 | fibrillin 2 | NM_001999.3 | 5q23.3 |
| FHL1 | four and a half LIM domains 1 | NM_00144.4 | Xq26 |
| FXN | frataxin | NM_000144.4 | 9q21.11 |
| GATA4 | GATA binding protein 4 | NM_002052.3 | 8p23.1-p22 |
| HCN4 | hyperpolarization activated cyclic nucleotide-gated potassium channel 4 | NM_005477.2 | 15q24.1 |
| ILK | integrin-linked kinase | NM_004517.3 | 11p15.4 |
| KCND3 | potassium voltage-gated channel, Shal-related subfamily, member 3 | NM_004980.4 | 1p13.3 |
| KCNE5 | KCNE1-like | NM_012282.2 | Xq22.3 |
| KCNE3 | potassium voltage-gated channel, Isk-related family, member 3 | NM_005472.4 | 11q13.4 |
| KCNJ5 | potassium inwardly-rectifying channel, subfamily J, member 5 | NM_000890.3 | 11q24 |
| KCNJ8 | potassium inwardly-rectifying channel, subfamily J, member 8 | NM_004982.2 | 12p11.23 |
| KLF10 | Kruppel-like factor 10 | NM_001032282.3 | 8q22.2 |
| LAMA4 | laminin, alpha 4 | NM_002290.4 | 6q21 |
| MYLK2 | myosin light chain kinase 2 | NM_033118.3 | 20q13.31 |
| MYOM1 | myomesin 1 | NM_003803.3 | 18p11.31 |
| MYPN | myopalladin | NM_032578.3 | 10q21.3 |
| NEBL | nebulette | NM_006393.2 | 10p12 |
| NKX2-5 | NK2 homeobox 5 | NM_004387.3 | 5q34 |
| NOTCH1 | notch 1 | NM_017617.3 | 9q34.3 |
| PDLIM3 | PDZ and LIM domain 3 | NM_014476.5 | 4q35 |
| PSEN1 | presenilin 1 | NM_000021.3 | 14q24.3 |
| PSEN2 | presenilin 2 | NM_000447.2 | 1q42.13 |
| PTPN11 | protein tyrosine phosphatase, non-receptor type 11 | NM_002834.3 | 12q24 |
| RAF1 | v-raf-1 murine leukemia viral oncogene homolog 1 | NM_002880.3 | 3p25 |
| RANGRF | RAN guanine nucleotide release factor | NM_016492.4 | 17p13.1 |
| SCN3B | sodium channel, voltage-gated, type III, beta subunit | NM_018400.3 | 11q23.3 |
| SLC8A1 | solute carrier family 8 (sodium/calcium exchanger), member 1 | NM_021097.2 | 2p22.1 |
| SMAD3 | SMAD family member 3 | NM_005902.3 | 15q22.33 |
| TBX1 | T-box 1 | NM_080647.1 | 22q11.21 |
| TBX5 | T-box 5 | NM_000192.3 | 12q24.1 |
| TGFBR1 | transforming growth factor, beta receptor 1 | NM_004312.2 | 9q22 |
| TRDN | triadin | NM_001251987 | 6q22.31 |
| TXNRD2 | thioredoxin reductase 2 | NM_006440.3 | 22q11.21 |

**Figure Legends (supplemental)**

**Figure S1,** *Diagnose of patients screened positive in a limited gene panel.*

**Figure S2,** *Variants discovered with our limited gene panels.*

**Figure S3,** *Coding effect of discovered variants in our limited gene panels. Genes where a small deletion was found are highlighted in the figure.*

**Figure S4,** *Pedigree of proband #1. BIV-ICD = Biventricular implantable cardio-defibrillator, HTX = Heart transplant, SCD = Sudden cardiac death*

**Figure S5,** *Pedigree of proband #5*

**Supplemental Figure S1**

**
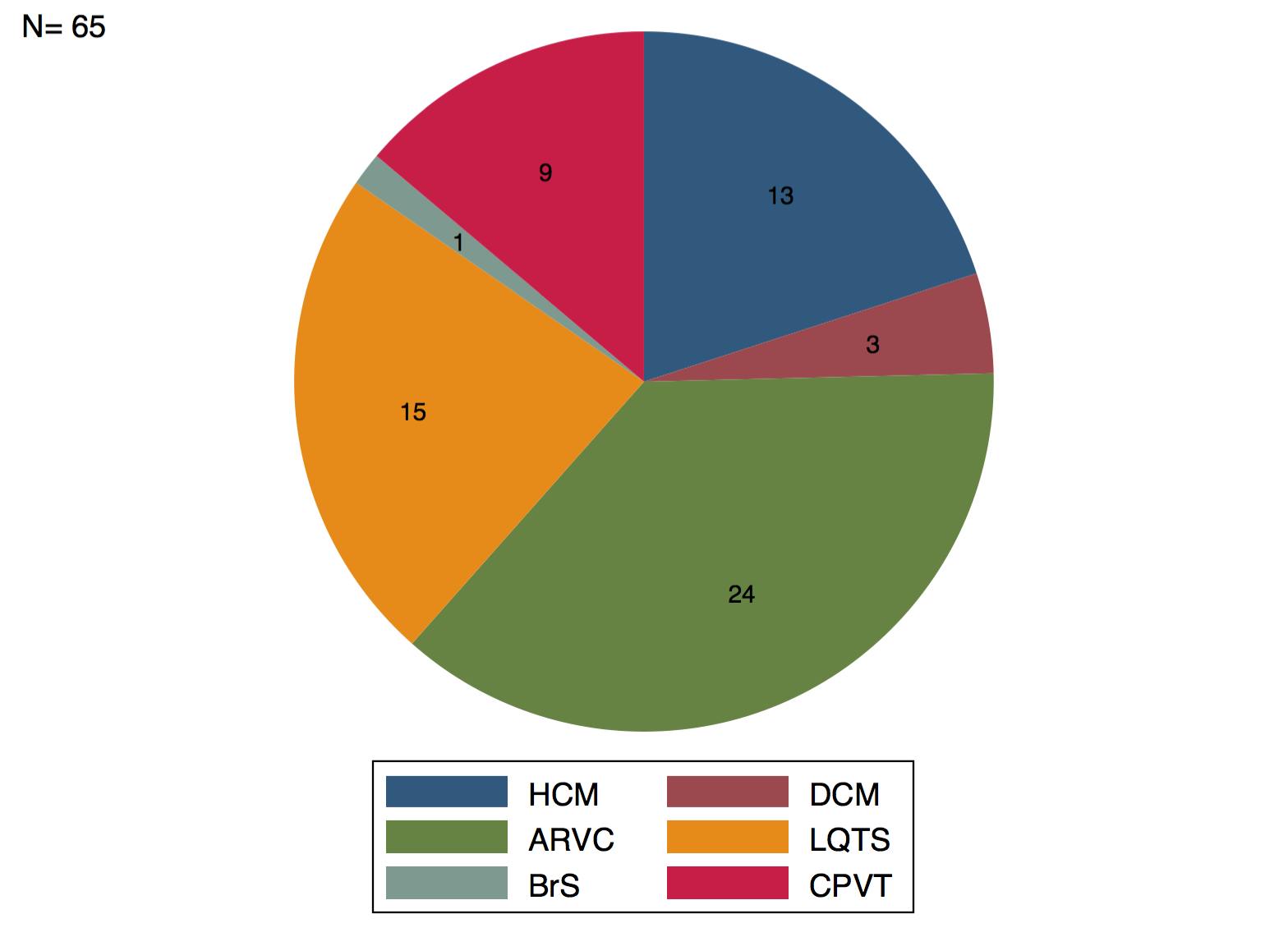
**

**Supplemental Figure S2**

**
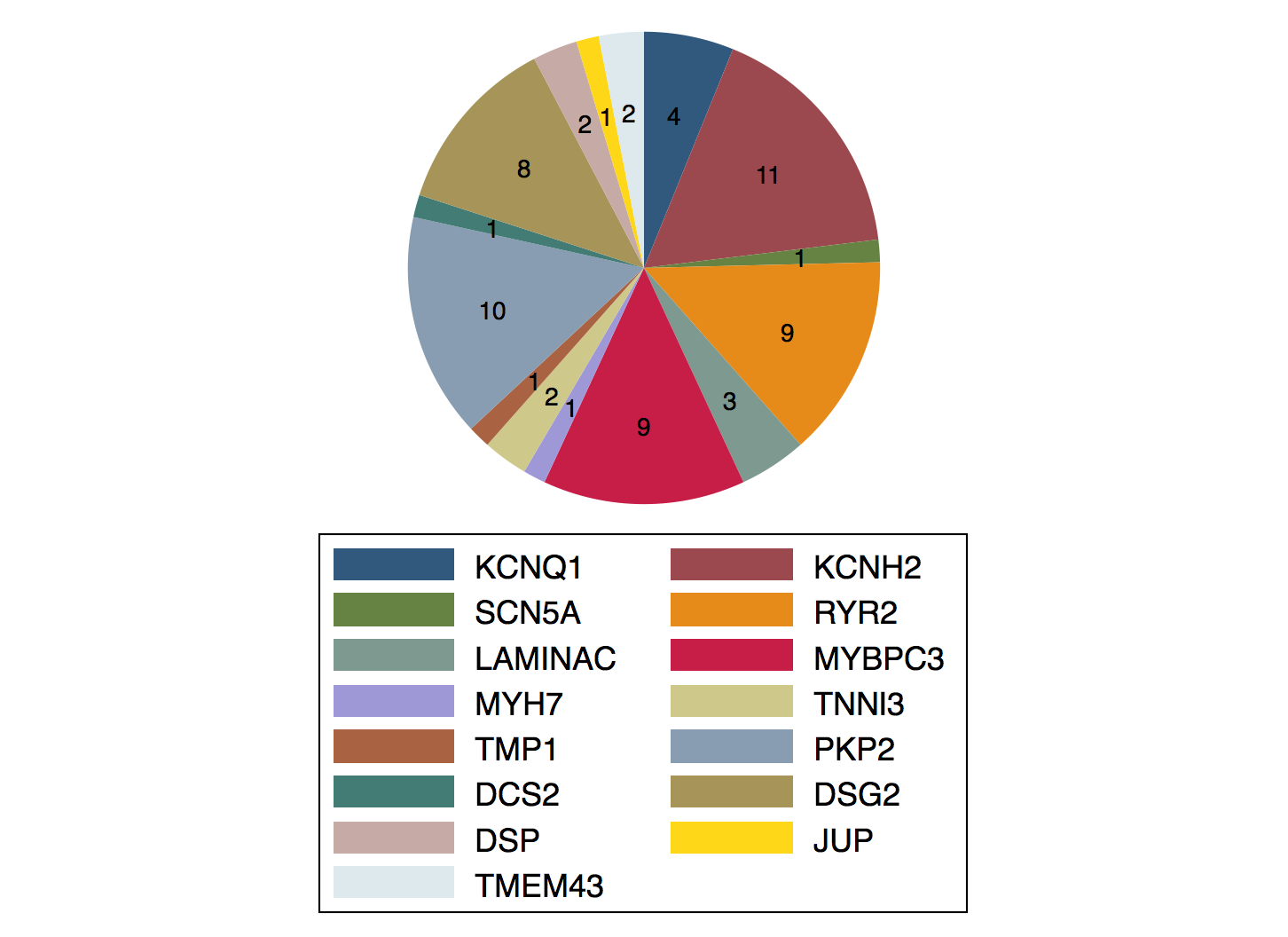
**

**Supplemental Figure S3**

**
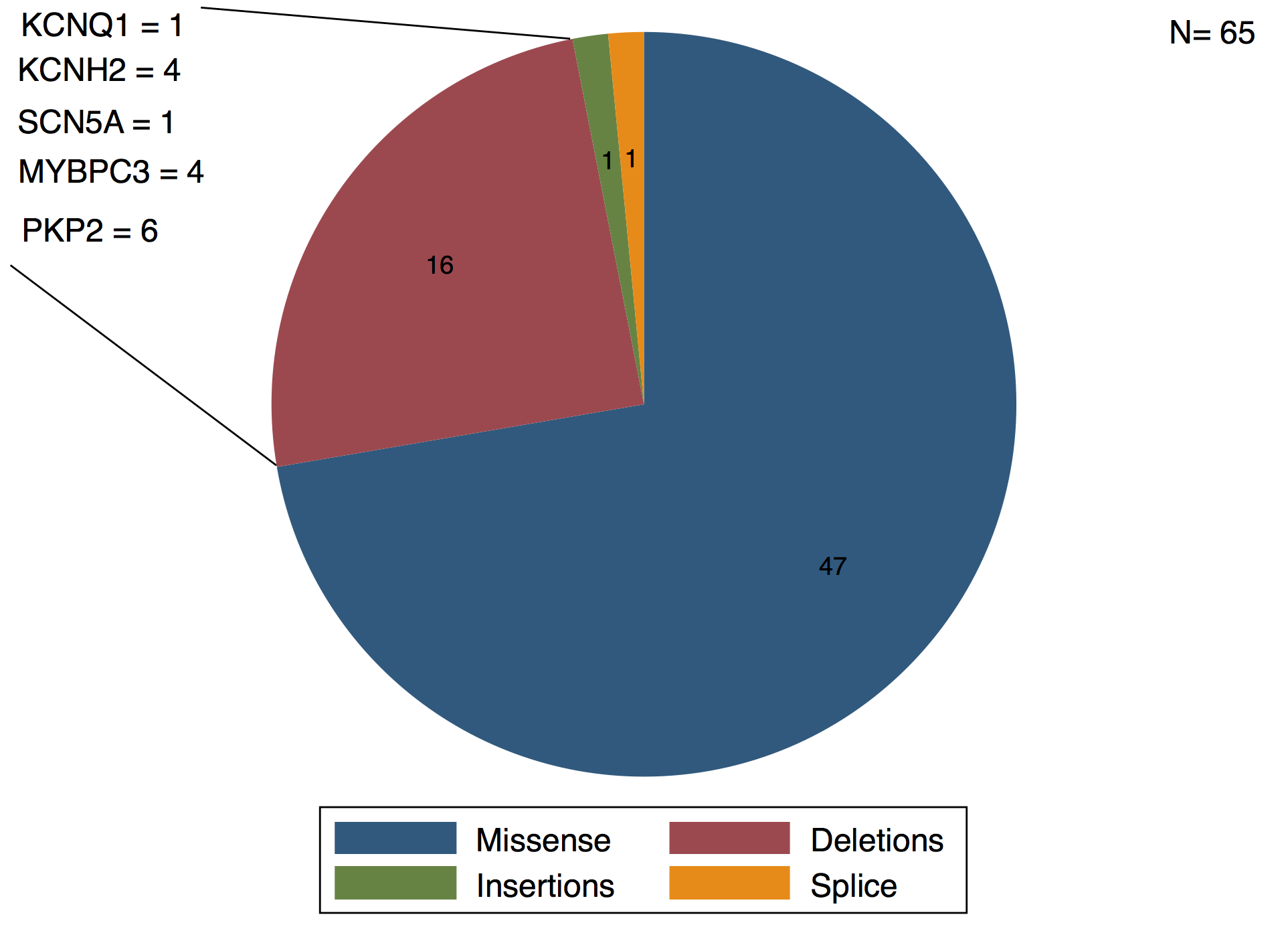
**

**Supplemental figure S4**

**
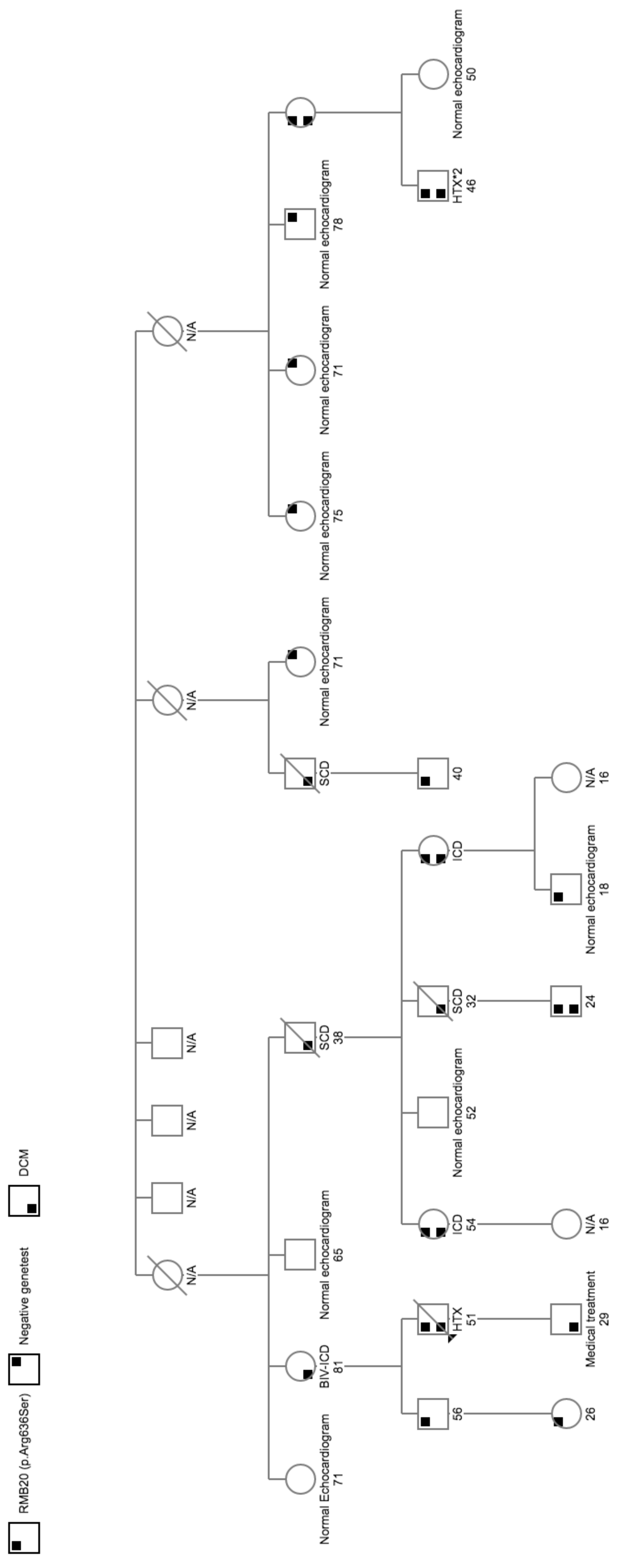
**

**Supplemental figure S5**

**
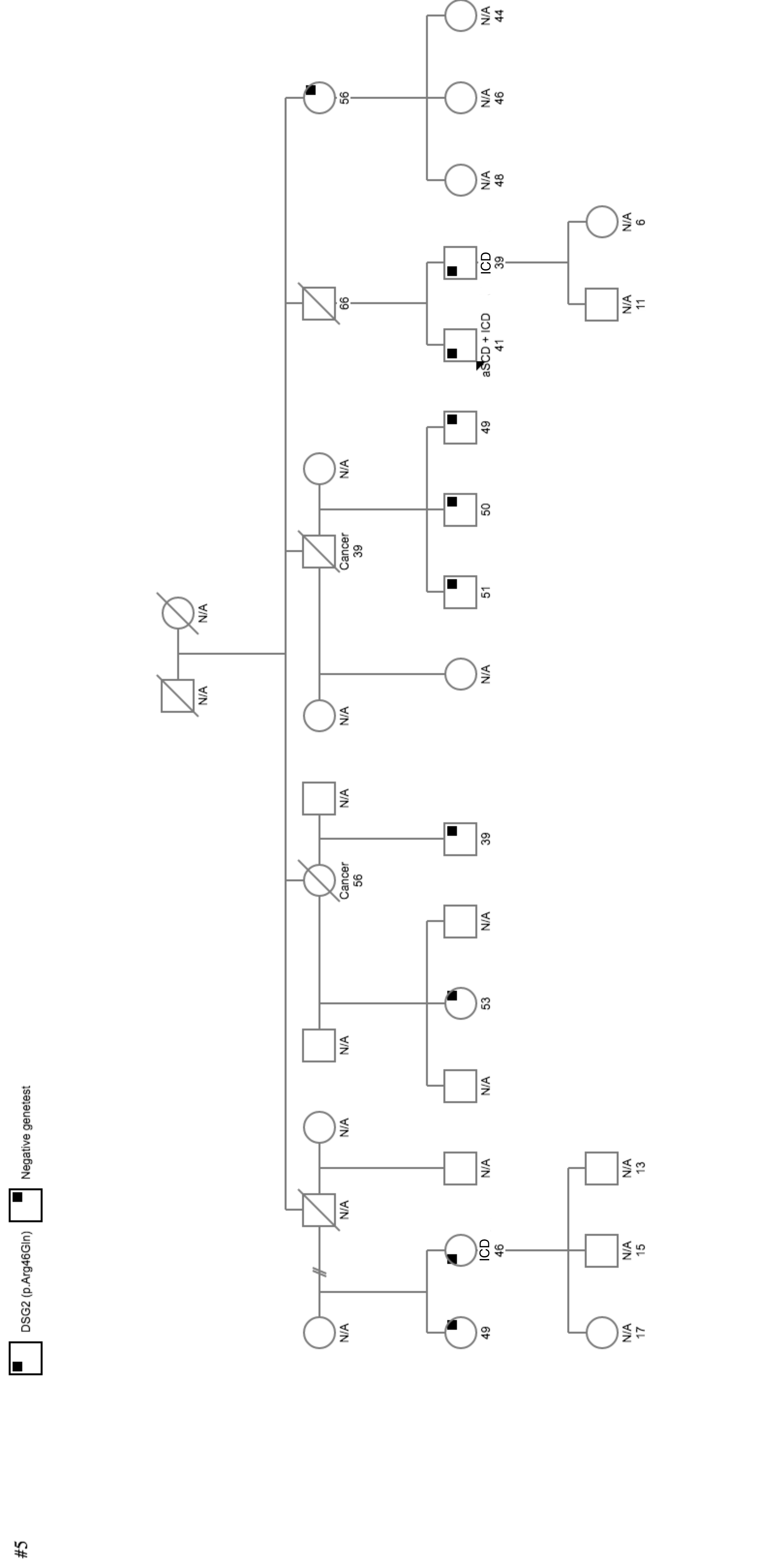
**

**LOVD database id’s**

Relevant variants have been submitted to the ARVC LOVD database with the submission IDs:

DSG2 arvcdatabase.info: DB:7538

DSC2 arvcdatabase.info: DB:7558 and DB:7632 Both not yet curated

<http://grenada.lumc.nl/LOVD2/ARVC>

Relevant variants have been submitted to the LOVD database curated by Zhejiang University Center for Genetic and Genomic Medicine with the submission IDs:

c.44 del, homozygote: CSRP3_00032

c.87C>A: KCNH2_00055

c.170T>C: KCNE2_00007

c.9854T>C: ANK2_00059

c.11791G>A: ANK2_00058

c.638G>A: SCN5A_00439

c.2291T>G: SCN5A_00438

All curated and published

<http://www.genomed.org/lovd2/home.php>

The following variants have been submitted to the LOVD database curated by Zhejiang University Center for Genetic and Genomic Medicine 2017-10-12:

c.4660G>C: MYH7_00305

c.345+1G>A: MYH7_00351

c.770C>G: SNTA1_00007

c.772G>A: c.MYBPC3_00010

c.170C>A: MYL3_00018

These five variants have not yet been curated.

**Proband and family history:**

*Pathogenic or likely pathogenic variants (ACMG score) in inherited cardiomyopathy*

*NEBL* (NM_006393.2):c.180G>C p.(Lys60Asn)

A 21-year old woman (#34, table 2) was admitted to the hospital with syncope and sustained VT. Clinical evaluation demonstrated normal echocardiography, CMR, exercise test and invasive electrophysiology study (EPS). Molecular genetic testing revealed a rare known *NEBL* variant. *In vivo* experiments with transgenic mice harbouring this variant has demonstrated development of DCM and severe heart failure.^2^ The *NEBL* variant was also found in her father, who has severe DCM with LVEDd of 75 mm and LVEF of 15%.

*MYBPC3* (NM_000256.3):c.772G>A p.(Glu258Lys)

A 41-year old man (#50, table 2) was admitted to hospital with dyspnoea. Telemetry demonstrated sustained monomorphic VT alternated with atrial fibrillation. Echocardiography showed a hypertrophic (septum 22 mm/posterior wall 13 mm) and dilated left ventricle (LVEDd =76 mm). Echocardiogram showed LVEF of 40% and an enlarged left atrium of 68 mm. Molecular genetic testing has revealed a known *MYBPC3* variant.^3^ The variant was also found in his brother, who has hypertrophic obstructive cardiomyopathy. Their father died suddenly at the age of 53 years. An autopsy of the father has not been performed.

*MYH7* splice variant

A 49-year old man (#62, table 2) collapsed with cardiac arrest and VF. Diagnostic examination with echocardiogram, CMR and exercise test was normal. No family history of SCD or cardiac disease. Molecular genetic testing revealed a rare novel splice variant at the donor site of intron 4, which makes a skip of exon 4 very likely.

*DSP* & *PKP* large genomic deletions

A 34-year old man (#23, table 2) was admitted with sustained VT. He had a dilated right ventricle (45 mm) by echocardiography and CMR. Positive signal averaged-electrocardiogram and biopsies showed fibro-fatty replacement. Molecular genetic testing revealed a large genomic *PKP2* deletion with an out of frame deletion of the entire exon 3 and part of the surrounding introns. Messenger RNA (mRNA) will probably be degraded to nonsense mediated RNA decay. His mother was clinically unaffected and harboured the same deletion.

A 37-year-old man (#59, table 2) had syncope and sustained VT as presenting symptom. Clinical evalution showed normal echocardiogram, CMR and signal averaged-electrocardiogram. However, molecular genetic testing revealed a deletion of exon 12, 13 and 14 in the *PKP2* gene. His mother does not harbor the deletion. His father died of old age.

A 42-year old woman (#79, table 2) was admitted with cardiac arrest and VF. After successful resuscitation, she had positive late potentials examination and Holter monitoring revealed 4000 premature ventricular contractions (PVC) over a period of 24 hours. She fulfilled the 2010 Task force criteria for arrhythmogenic right ventricular cardiomyopathy (ARVC) due to a positive family history, frequent PVCs and positive late potentials. Surprisingly, her echocardiography and CMR investigation were both normal. Molecular genetic testing revealed a novel large genomic deletion in the *DSP* gene (exon 3-15) with possible haploinsufficiency as a consequence. Her father died at the age of 51 years. Her mother died of cancer at the age of 52 years. A clinical examination of her oldest brother including an ECG, an echocardiography, a signal-averaged electrocardiogram and a CMR were all normal, despite the fact that he harbors the same deletion. He was offered a primary prophylactic ICD. Her youngest brother died suddenly at the age of 41 years, however, autopsy and genetic testing has not been performed.

*Pathogenic or likely pathogenic variants (ACMG score) in inherited ion channel disease/idiopathic ventricular fibrillation:*

*SNTA1* (NM_003098.2): c.770C>G p.(Ala257Gly)

A 12-year old girl (#20, table 2) was admitted to the hospital with cardiac arrest and VF. ECG demonstrated a normal QTc interval. Molecular genetic testing revealed a rare *SNTA1* (NM_003098.2): c.770C>G (p.Arg257Gly) variant. In vivo studies have shown that this *SNTA1* variant can cause a gain-of-function of the Nav1.5.^4^ The *SNTA1* (NM_003098.2): c.221C>T (p.Pro74Leu) variant was also found. The (p.Pro74Leu) variant has been demonstrated to have a rescue function when present in conjunction with (p.Arg257Gly) . This variant combination reversed the peak sodium current and window current induced by the (p.Arg257Gly) variant alone.^5^ However, it seems that the (p.Pro74Leu) variant did not have the described rescue effect in the present case.

*KCNH2* (NM_000238.2):c.87C>A p.(Phe29Leu)

A 17-year old man (#63, table 2) was admitted to cardiac care with recurrent syncope and VT. His ECG demonstrated a normal QTc interval. Molecular genetic testing revealed a known genetic variant in *KCNH2*. His brother and his mother had prolonged QTc in the ECG (460-470 ms) but were asymptomatic. Both were carriers of the same genetic variant. Two family members had SCD at young age and a cousin had aSCD at the age of 10. Clinically we have a strong suspicion of hereditary LQTS with reduced penetrance in the family.

*RYR2* (NM_001035.2)c.14553C>A p.(Phe4851Leu)

A 10-year old boy (#75, table 2) suffered from cardiac arrest and VF. He had a normal ECG and echocardiography, but suffered from post resuscitation brain damage. He has severe multifocal atrial tachycardia, which caused recurrent inappropriate shock episodes. Molecular genetic testing revealed a rare known *RYR2* variant.^6^ Neither of the parents harboured the variant.

*RYR2* (NM_001035.2)c.1258C>T p.(Arg420Trp)/ *RYR2* (NM_001035.2)c.3407C>T p.(Ala1136Val)

A 33-year old woman (#76, table 2 & 3) was admitted with cardiac arrest and VF. She had a normal ECG and echocardiography. Molecular genetic screening revealed two different known *RYR2* variants. ^7,8^ The proband’s mother and maternal uncle suffered from SCD at the age of 26. Post mortem genetic testing has not been possible due to tissue/blood have not been preserved. The proband’s children have not responded to enquiry of clinical and genetic examination.

*SCN5A* (NM_198056.2)c.638G>A p.(Gly213Asp)

A 42-year old woman (#77, table 2) presented with syncope and sustained VT. ECG demonstrated rare sinus beats interpolated between frequent premature atrial and ventricular beats. She had a normal CMR without late gadolinium enhancement. Genetic screening revealed a very rare *SCN5A* variant. Fourteen relatives carried the variant and thirteen relatives presented with a similar phenotype. Additionally three family members had the same phenotype but did not consent for genotyping.

*CSRP3* (NM_003476.3)c.44del p.(Lys15fs)/ *MYL3* (NM_000258.2)c.170C>A p.(Ala57Asp)

/*KCNE2* (NM_172201.1)c.170T>C p.(Ile57Thr)

An 18-year old girl (#78, table 2) was admitted after witnessed cardiac arrest. Her primary rhythm was VF. Her ECG was normal. Echocardiography showed normal systolic function. CMR with late gadolineum enhancement showed no sign of fibrosis. Molecular genetic screening revealed a novel homozygote *CRSP3* one base deletion, which causes frameshift and premature stop at the codon 192 amino-acids downstream. A rare *KCNE2* variant was also found. This variant has been associated with gain of function of the transient outward current of the potassium channel. *KCNE2* plays a critical role in normal ion channel function.^9^ We also found a rare *MYL3* variant. *In vivo* and *in vitro* models have demonstrated high level of fibrosis, hypertrophy and enhanced myocardial stiffness.^10^

**References (Supplemental)**

1. Brauch KM, Karst ML, Herron KJ, et al. Mutations in ribonucleic acid binding protein gene cause familial dilated cardiomyopathy. *Journal of the American College of Cardiology*. 2009;54(10):930-941. doi:10.1016/j.jacc.2009.05.038.

2. Purevjav E, Varela J, Morgado M, et al. Nebulette mutations are associated with dilated cardiomyopathy and endocardial fibroelastosis. *Journal of the American College of Cardiology*. 2010;56(18):1493-1502. doi:10.1016/j.jacc.2010.05.045.

3. Vignier N, Schlossarek S, Fraysse B, et al. Nonsense-mediated mRNA decay and ubiquitin-proteasome system regulate cardiac myosin-binding protein C mutant levels in cardiomyopathic mice. *Circulation Research*. 2009;105(3):239-248. doi:10.1161/CIRCRESAHA.109.201251.

4. Wu G, Ai T, Kim JJ, et al. alpha-1-syntrophin mutation and the long-QT syndrome: a disease of sodium channel disruption. *Circulation: Arrhythmia and Electrophysiology*. 2008;1(3):193-201. doi:10.1161/CIRCEP.108.769224.

5. Cheng J, Norstrand DWV, Medeiros-Domingo A, et al. LQTS-associated mutation A257G in α1-syntrophin interacts with the intragenic variant P74L to modify its biophysical phenotype. *Cardiogenetics*. 2011;1(1).

6. Hayashi M, Denjoy I, Extramiana F, et al. Incidence and Risk Factors of Arrhythmic Events in Catecholaminergic Polymorphic Ventricular Tachycardia. *Circulation*. 2009;119(18):2426-2434. doi:10.1161/CIRCULATIONAHA.108.829267.

7. Bauce B, Rampazzo A, Basso C, et al. Screening for ryanodine receptor type 2 mutations in families with effort-induced polymorphic ventricular arrhythmias and sudden death. *Journal of the American College of Cardiology*. 2002;40(2):341-349. doi:10.1016/S0735-1097(02)01946-0.

8. Krahn AD, Healey JS, Chauhan V, et al. Systematic Assessment of Patients With Unexplained Cardiac Arrest: Cardiac Arrest Survivors With Preserved Ejection Fraction Registry (CASPER). *Circulation*. 2009;120(4):278-285. doi:10.1161/CIRCULATIONAHA.109.853143.

9. Wu J, Shimizu W, Ding W-G, et al. KCNE2 modulation of Kv4.3 current and its potential role in fatal rhythm disorders. *Heart Rhythm*. 2010;7(2):199-205. doi:10.1016/j.hrthm.2009.10.012.

10. Kazmierczak K, Paulino EC, Huang W, et al. Discrete effects of A57G-myosin essential light chain mutation associated with familial hypertrophic cardiomyopathy. *Am J Physiol Heart Circ Physiol*. 2013;305(4):H575-H589. doi:10.1152/ajpheart.00107.2013.

1. Contains MOMA heart panel v1 (except KCNQ4) and genes mentioned in table S2 [↑](#footnote-ref-1)
